# Supplementary material for: Risk Factors for the Development of Nontuberculous Mycobacteria Pulmonary Disease during Long-Term Follow-Up after Lung Cancer Surgery
Source: Diagnostics (Basel). 2022 Apr 27;12(5):1086. doi: 10.3390/diagnostics12051086 (PMC9139784; doi:10.3390/diagnostics12051086)
Supplement: Supplementary file 1 [file diagnostics-12-01086-s001.zip › diagnostics-1633826-SI.pdf]

Table S1. The baseline characteristics of patients with NSCLC and the development of NTM-positive results after lung resection

Variables	NTM-positive*		
	NTM (–) (n = 6347)	NTM (+) (n = 156)	*P*
Age, years	63 (56–69)	65 (59–70)	0.007
Age > 65 years	2703 (42.6)	84 (53.8)	0.005
Sex, male	3864 (60.9)	115 (73.7)	0.001
Smoking status (n = 6501)			0.002
Never smoker	2703 (42.6)	46 (29.5)	
Ex-smoker	1986 (31.3)	54 (34.6)	
Current smoker	1656 (26.1)	56 (35.9)	
Pack-years (n = 3761)	30 (20–45)	40 (30–52)	0.001
BMI, kg/m^2	23.9 (22.0–25.8)	23.3 (20.9–25.1)	<0.001
BMI ≤ 18.5kg/ m^2	163 (2.6)	10 (6.4)	0.008
Comorbidity			
Pulmonary disease			
History of pulmonary TB	666 (10.5)	35 (22.4)	<0.001
COPD/Asthma	1697 (26.7)	62 (39.7)	<0.001
Interstitial lung disease	70 (1.1)	4 (2.6)	0.101
DM	998 (15.7)	23 (14.7)	0.739
Hypertension	2300 (36.2)	63 (40.4)	0.287
Chronic heart disease	441 (6.9)	10 (6.4)	0.794
Chronic renal disease	88 (1.4)	1 (0.6)	0.726
Cerebrovascular disease	372 (5.9)	3 (1.9)	0.037
Previous malignancy	877 (13.8)	22 (14.1)	0.919
Clinical stage at diagnosis			0.001†
Stage I	4395 (69.2)	92 (59.0)	
Stage II	1101 (17.3)	28 (17.9)	
Stage III	791 (12.5)	35 (22.4)	
Stage IV	60 (0.9)	1 (0.6)	
Tumor histology			0.046
Adenocarcinoma	4505 (71.0)	97 (62.2)	
Squamous cell carcinoma	1464 (23.1)	45 (28.8)	
Others‡	378 (6.0)	14 (9.0)	
Location of lung cancer			0.115
Right	3668 (57.8)	100 (64.1)	
Left	2679 (42.2)	56 (35.9)	
CT findings			
TB sequelae	266 (4.2)	11 (7.1)	0.081
Bronchiectasis	378 (6.0)	24 (15.4)	<0.001
Centrilobular bronchiolitis	137 (2.2)	19 (12.2)	<0.001

Data are presented as n (%) or the median (interquartile range). NSCLC, non-small cell lung cancer; NTM, nontuberculous mycobacteria; BMI, body mass index; TB, tuberculosis; COPD, chronic obstructive pulmonary disease; DM, diabetes mellitus. *The sum of confirmed and suspected patients with NTM-PD. †P values were calculated with the use of a Mantel-Haenszel test (trend test). ‡Includes large cell neuroendocrine carcinoma, adenosquamous carcinoma, pleomorphic carcinoma, adenoid cystic carcinoma, mucoepidermoid carcinoma, epithelial myoepithelial carcinoma, and carcinoid tumor.

Table S2. The treatment profile for NSCLC and the development of NTM-positive results after lung resection

**Variables**	**NTM-positive***		
	NTM (–) (n = 6347)	NTM (+) (n = 156)	*P*
Neoadjuvant treatment			
No	5752 (90.6)	127 (81.4)	<0.001
Yes	595 (9.4)	29 (18.6)	
CCRT	515 (8.1)	26 (16.7)	<0.001
Chemotherapy	74 (1.2)	2 (1.3)	0.705
Radiotherapy	6 (0.1)	1 (0.6)	0.156
Surgical approach			<0.001
VATS	3982 (62.7)	72 (46.2)	
Thoracotomy	2365 (37.3)	84 (53.8)	
Extent of surgical resection			0.553†
Sublobar resection	1070 (16.8)	18 (11.5)	
Wedge resection	623 (9.8)	11 (7.0)	0.805
Segmentectomy	447 (7.0)	7 (4.5)	
Lobectomy	4817 (75.9)	124 (79.5)	
Bilobectomy	238 (3.7)	11 (7.1)	
Pneumonectomy	222 (3.5)	3 (1.9)	
Pathologic stage‡			0.088†
I	4054 (64.5)	91 (60.5)	
II	1181 (18.8)	24 (15.8)	
III	981 (15.6)	34 (22.4)	
IV	73 (1.2)	2 (1.3)	
PPC§	1047 (16.5)	51 (32.7)	<0.001
Adjuvant treatment¶			0.018
No	4577 (72.1)	100 (64.1)	
Yes	1722 (27.9)	56 (35.9)	
CCRT	321 (5.1)	8 (5.1)	0.968
Chemotherapy	1108 (17.5)	32 (20.5)	0.321
Radiotherapy	293 (4.6)	16 (10.3)	0.001

Data are presented as n (%). NSCLC, non-small cell lung cancer; NTM, nontuberculous mycobacteria; CCRT, concurrent chemoradiotherapy; VATS, video-assisted thoracoscopic surgery; PPC, postoperative pulmonary complication. *The sum of confirmed NTM-PD and suspicious NTM-PD patients. †P values were calculated with the use of a Mantel-Haenszel test (trend test). ‡Except for 63 patients where no re-sidual tumor appeared in the surgical specimen after neoadjuvant treatment (pathologic complete response [ypCR]). §Pneumothorax and/or prolonged air leak (n = 502), respiratory failure that required mechanical ventilation (n = 253), pneumonia (n = 231), pleural effusion (n = 150), others (atelectasis, bronchopleural fistula, pulmonary thromboembolism, etc.) (n = 291). Some patients had more than one complication. ¶Excluded 48 patients due to data unavailability.

Table S3. Characteristics of suspicious NTM-PD

Variables	n (%)
**Suspicious NTM-PD (n = 97)**	
NTM culture (+) from respiratory specimen without identification test for NTM species	68 (70.1)
NTM culture (+) from only one sputum sample with identification test for NTM species	25 (25.8)
M. avium	6 (6.2)
M. intracellulare	6 (6.2)
M. fortuitum complex	6 (6.2)
M. abscessus	3 (3.1)
Others*	4 (4.1)
NTM culture (+) from at least two sputum samples with only one identification test for NTM species	4 (4.1)
M. avium	2 (2.1)
M. intracellulare	1 (1.0)
M. massiliense	1 (1.0)
Radiologic findings	
Nodular bronchiectatic form	79 (81.4)
Without cavity	75 (77.3)
With cavity	4 (4.1)
Fibrocavitary form	18 (18.6)

Data are presented as n (%). NTM-PD, nontuberculous mycobacteria pulmonary disease. *M. lentiflavum/M. genavense (n = 2), M. terrae complex (n = 1), and M. gordonae (n = 1). M. lentiflavum/M. genavense was reported together because the kit we used for testing could not dif-ferentiate between these two species.

Table S4. Prognostic factors associated with the development of NTM-positive results after lung resection for NSCLC (N = 6503)

Variables	NTM-positive*			
	Univariable Cox		Multivariable Cox	
	Unadjusted HR (95% CI)	*P*	Adjusted HR (95% CI)	*P*
Host-related factors				
Age > 65 years	2.07 (1.50-2.83)	<0.001	1.74 (1.26-2.41)	0.001
Sex, male	2.12 (1.49-3.04)	<0.001		
BMI ≤ 18.5 kg/m^2	3.23 (1.70-6.13)	<0.001		
Smoking history, yes	2.10 (1.49-2.96)	<0.001	1.48 (1.02-2.13)	0.037
Comorbidity				
History of pulmonary TB	2.55 (1.75-3.71)	<0.001	2.27 (1.55-3.31)	<0.001
COPD/Asthma	2.02 (1.47-2.79)	<0.001		
ILD	4.90 (1.81-13.25)	0.002	4.22 (1.54-11.56)	0.005
Diabetes mellitus	1.05 (0.68-1.64)	0.817		
History of malignancy	1.04 (0.66-1.63)	0.877		
CT findings				
TB sequelae	1.80 (0.98-3.33)	0.060		
Bronchiectasis	2.67 (1.73-4.13)	<0.001	2.28 (1.62-3.21)	0.002
Centrilobular bronchiolitis	6.09 (3.77-9.84)	<0.001	4.42 (2.65-7.39)	<0.001
Cancer-related factors				
Tumor histology				
Adenocarcinoma	Reference			
Squamous cell carcinoma	1.83 (1.28-2.61)	0.001		
Others†	2.18 (1.24-3.81)	0.007		
Treatment-related factors				
Surgical approach				
VATS	Reference		Reference	
Thoracotomy	2.52 (1.84-3.46)	<0.001	1.53 (1.07-2.21)	0.021
Extent of surgical resection				
Lobectomy	Reference			
Sublobar resection	0.64 (0.39-1.04)	0.073		
Bilobectomy	2.09 (1.13-3.87)	0.019		
Pneumonectomy	0.75 (0.24-2.35)	0.617		
PPC‡	2.93 (2.10-4.10)	<0.001	2.28 (1.62-3.21)	<0.001
Neoadjuvant and adjuvant treatment				
No	Reference		Reference	
CTx or RTx alone	1.35 (0.89-2.04)	0.155	1.18 (0.76-1.82)	0.464
CTx and RTx both	2.40 (1.63-3.55)	<0.001	2.13 (1.38-3.29)	0.001

NTM, nontuberculous mycobacteria; NSCLC, non-small cell lung cancer; HR, hazard ratio; CI, confiden-tial interval; BMI, body mass index; TB, tuberculosis; COPD, chronic obstructive pulmonary disease; ILD, interstitial lung disease; CT, computed tomography; VATS, video-assisted thoracoscopic surgery; PPC, postoperative pulmonary complication; CTx, chemotherapy; RTx, radiotherapy. *The sum of confirmed NTM-PD and suspicious NTM-PD patients. †Includes large cell neuro-endocrine carcinoma, adenosquamous carcinoma, pleomorphic carcinoma, adenoid cystic carcinoma, mucoepidermoid carcino-ma, epithelial myoepithelial carcinoma, and carcinoid tumor. ‡Pneumothorax and/or prolonged air leak (n = 502), respiratory failure that required mechanical ventilation (n = 253), pneumonia (n = 231), pleural effusion (n = 150), others (atelectasis, bron-chopleural fistula, pulmonary thromboembolism, etc.) (n = 291). Some patients had more than one complication
